# Supplementary material for: Epidemiological, clinical and laboratory profile of patients presenting with severe acute respiratory syndrome (SARS-CoV-2) in Ethiopia
Source: PLoS One. 2023 Dec 1;18(12):e0295177. doi: 10.1371/journal.pone.0295177 (PMC10691732; doi:10.1371/journal.pone.0295177)
Supplement: S1 Checklist — (DOCX) [file pone.0295177.s001.docx]

STROBE Statement—checklist of items that should be included in reports of observational studies

|  | | | | Item No. | | Recommendation | | Page  No. | | | | | | | Relevant text from manuscript | | | | | | | | | | | | | | | | | | | | | | | | | | | | | |
| --- | --- | --- | --- | --- | --- | --- | --- | --- | --- | --- | --- | --- | --- | --- | --- | --- | --- | --- | --- | --- | --- | --- | --- | --- | --- | --- | --- | --- | --- | --- | --- | --- | --- | --- | --- | --- | --- | --- | --- | --- | --- | --- | --- | --- |
| **Title and abstract** | | | | 1 | | (*a*) Indicate the study’s design with a commonly used term in the title or the abstract | | 1 | | | | | | | Comparative cross-sectional study | | | | | | | | | | | | | | | | | | | | | | | | |  |  |  |  |  |
|  |  |  |  |  |  | (*b*) Provide in the abstract an informative and balanced summary of what was done and what was found | | 1 | | | | | | | The study aimed to compare epidemiological, clinical and laboratory profiles patients presenting with acute respiratory syndrome illness in Addis Ababa Ethiopia. We found that patients with COVID-19 have significantly higher Neutrophil, Creatinine, and Alkaline phosphatase significantly higher in the COVID-19 patients than non-infected patients. This finding suggests that the need to substantially consider headache, sore throat, and loss of taste as potential clinical diagnostic symptoms for early screening and testing in the country’s context and Neutrophil, Creatinine, Alkaline phosphatase profiles may also be potential diagnostic biomarkers in screening and testing for suspected patients. | | | | | | | | | | | | | | | | | | | | | | | |  |  |  |  |  |  |
| Introduction | | | | | | | | | | | | | | |  | | | | | | | | | | | | | | | | | | | | | | | | | | | | | |
| Background/rationale | | | | 2 | | Explain the scientific background and rationale for the investigation being reported | | 3, 4 | | | | | | | COVID-19 is caused by SARS-CoV-2, still a global concern has the clinical spectrum ranges from asymptomatic or mild to severe life-threatening conditions. However, most of the clinical pictures presented by symptomatic patients are similar to other respiratory disease manifestations. The majority of the global studies described from patients of high-income settings. However, patients managed at low resource settings might be quite different from what was described because of socio-demographic, quality of medical care, nutrition, and genetic and immunological factors. In addition, as to our knowledge analogous data have not been previously published in a peer-reviewed journal and we believe the conclusions provide distinct insights that are of relevance to a similar context. Furthermore, the continued emergence of new variants of SARS- CoV-2 is also associated with a change in the clinical presentations of the disease, which requires monitoring of the clinical profile of patients.  Thus, this study aimed to generate evidence about the most pertinent epidemiologic features, clinical profiles and laboratory findings COVID-19 patients managed in low-resource settings like in Ethiopia to guide the testing and treatment strategies. | | | | | | | | | | | | | | | | | | | |  |  |  |  |  |  |  |  |  |  |
| Objectives | | | | 3 | | State specific objectives, including any prespecified hypotheses | | 4 | | | | | | | To generate evidence about the most pertinent epidemiologic features.  To characterize clinical profiles patients with SARS-CoV-2 infected and suspected patients  To compare the laboratory findings SARS-CoV-2 infected patients from the non-infected symptomatic participants | | | | | | | | | | | | | | | | | | | | | | | | |  |  |  |  |  |
| Methods | | | | | | | | | | | | | | |  | | | | | | | | | | | | | | | | | | | | | | | | | | | | | |
| Study design | | | | 4 | | Present key elements of study design early in the paper | | 5 | | | | | | | Comparative cross-sectional study was conducted for all SARS-CoV-2 symptomatic ill patients. | | | | | | | | | | | | | | | | | | | | | | | | | |  |  |  |  |
| Setting | | | | 5 | | Describe the setting, locations, and relevant dates, including periods of recruitment, exposure, follow-up, and data collection | | 4, 5 | | | | | | | The study was conducted on one of main COVID-19 centre of the country, SPHMMC in Addis Ababa, Ethiopia during the SARS-CoV-2 infected patients and those without the disease who are presenting respiratory illness, for a year, from 1 October 2020 to 16 September 2021. Patients presenting with SARS-CoV-2 came to the centre from various corners of the country outside of the capital city of Ethiopia.  We identified all patients 18 years above who underwent testing for SARS-CoV-2 within 24 hours of presentation to this referral hospital. Suspected patients were tested for SARS-CoV-2 whether they met or not the COVID-19 clinical and laboratory testing criteria of the National Ethiopian Public Health Guideline.  The study included any acute respiratory illness (runny nose and sore throat) and at least one of the following symptoms: fever, cough, and shortness of breath.  A pre-tested questionnaire and check-list were used to collect socio-demographic status and clinical and laboratory tests of the study participants. The data was collected by trained physicians and laboratory technologists, and all authors had no an access to information that could identify individual participants during or after data collection. Accordingly, patient clinical data on initial clinical presentation, comorbidities, and relevant treatment and clinical outcomes for all patients presented with acute respiratory symptoms or influenza-like illness symptoms were recorded using the prepared formats for each department. | | | | | | | | | | | | | | | | | | | | | | | | | | |  |  |  |
| Participants | | | | 6 | | (*a*) *Cohort study*—Give the eligibility criteria, and the sources and methods of selection of participants. Describe methods of follow-up  *Case-control study*—Give the eligibility criteria, and the sources and methods of case ascertainment and control selection. Give the rationale for the choice of cases and controls  *Cross-sectional study*—Give the eligibility criteria, and the sources and methods of selection of participants | | 5 | | | | | | | All suspected COVID-19 patients who have essential clinical features of acute respiratory illnesses or the most common presenting symptoms of acute respiratory illnesses (cough and dyspnea**),** influenza-like symptoms (fever and myalgia) and known contact history with COVID-19 patients were included in the study from the isolation centre, whereas previously known COVID-19 disease patients were excluded from the study. | | | | | | | | | | | | | | | | | | | | | | | | |  |  |  |  |  |
|  |  |  |  |  |  | (*b*) *Cohort study*—For matched studies, give matching criteria and number of exposed and unexposed  *Case-control study*—For matched studies, give matching criteria and the number of controls per case | |  | | | | | | |  | | | | | | | | | | | | | | | | | | | | | | | | | | | | | |
| Variables | | | | 7 | | Clearly define all outcomes, exposures, predictors, potential confounders, and effect modifiers. Give diagnostic criteria, if applicable | | 5 | | | | | | | We were comparing SARS-CoV-2 infected patients and those without the disease who are presenting respiratory illness. Therefore, our outcome variables were epidemiological characteristics, clinical profile of infected and non-infected patients, and laboratory profile of SARS-CoV-2 infected and non-infected patients. The predictors were socio-demographic factors, presence and absence of SARS-CoV-2, essential clinical features of acute respiratory illnesses or the most common presenting symptoms of acute respiratory illnesses (cough and dyspnea**),** influenza-like symptoms (fever and myalgia) and known contact history with COVID-19, preventive measures of SARS-CoV-2, and some of the comorbid disease. | | | | | | | | | | | | | | | | | | | | | | | | | | | | |  |
| Data sources/ measurement | | | | 8* | | For each variable of interest, give sources of data and details of methods of assessment (measurement). Describe comparability of assessment methods if there is more than one group | | 4,5,6,7 | | | | | | | We identified all patients 18 years above who underwent testing for SARS-CoV-2 within 24 hours of presentation to this referral hospital. After the study subjects met the COVID-19 clinical and microbiological laboratory testing criteria of the Ethiopian Public Health Institute Guideline (EPHI), oropharyngeal (OP) and/or nasopharyngeal (NP) swabs were collected to confirm the disease using RT-PCR assay at the SPHMMC testing centre.  Whole blood samples were collected aseptically from each study subject, fresh (˂4 hours from collection) dipotassium EDTA–anticoagulated collected in Vacutainer tubes (Becton Dickinson), and run Beckman Coulter DxH 800 Hematology Analyzer (California, USA).  The collected and coagulated blood was used for enzymatic and biochemical tests for liver and renal function assessment at the chemistry laboratory using Roche Cobas C 501 Chemistry Analyzer at the clinical chemistry laboratory. Then suspected patients were tested for SARS-CoV-2 whether they met or not the COVID-19 clinical and laboratory testing criteria of the National Ethiopian Public Health Guideline.  Finally, infected patient with SARS-CoV-2 clinical, laboratory data were compared with suspected one but not infected patients. | | | | | | | | | | | | | | | | | | | | | | | | | | | |  |  |
| Bias | | | | 9 | | Describe any efforts to address potential sources of bias | | 1,4&5 | | | | | | | We identified all patients 18 years above who underwent testing for SARS-CoV-2 within 24 hours of presentation to this referral hospital.  The study included any acute respiratory illness (runny nose and sore throat) and at least one of the following symptoms  Comparative cross-sectional study design among patients with SARS-CoV-2 illness, then we used structured questionnaire a consecutive sampling technique was applied. | | | | | | | | | | | | | | | | | | | | | | | | | | | |  |  |
| Study size | | | | 10 | | Explain how the study size was arrived at | | 5 | | | | | | | The sample size used for this study was based on the proportion of patients with COVID-19 positivity status. Because we had no previous proportion of positivity during the study period, we took the positivity rate of RT-PCR COVID-19 to be 50%, with a 5% margin of error, a 95% confidence level, and a 10% non-response rate and keeping the assumption of a single population proportion formula. Therefore, the final sample size was determined to be 422 study subjects. | | | | | | | | | | | | | | | | | | | | | | | |  |  |  |  |  |  |
| Continued on next page Quantitative variables | | 11 | | | Explain how quantitative variables were handled in the analyses.  If applicable, describe which groupings were chosen and why | | | 7&8 | | | | | | | A descriptive analysis was done to see the characteristics of the study subjects. Continuous variables were dichotomized when clinically relevant. We estimated the mean and standard deviation for normally distributed continuous variables and used t-tests to see if there were any differences between the COVID-19 positive and COVID-19 negative patients. | | | | | | | | | | | | | | | | | | | | | | |  |  |  |  |  |  |  |
| Statistical methods | | 12 | | | (*a*) Describe all statistical methods, including those used to control for confounding | | 7&8 | | | | | | | | We used the chi-square test or Fisher's exact test to assess differences between groups for categorical and dichotomous data. The statistical significance was tested at a 95% CI and p ≤ 0.05 was considered statistically significant. | | | | | | | | | | | | | | | | | | | | | |  |  |  |  |  |  |  |  |
|  |  |  |  |  | (*b*) Describe any methods used to examine subgroups and interactions | | | | | | | | | | | | | | | | | | | | | | | |  | | |  | | | | | | |  |  |  |  |  |  |
|  |  |  |  |  | (*c*) Explain how missing data were addressed | | | | | | | | | | | | | | | | | | | | | | | |  | | |  | | | | | | |  |  |  |  |  |  |
|  |  |  |  |  | (*d*) *Cohort study*—If applicable, explain how loss to follow-up was addressed  *Case-control study*—If applicable, explain how matching of cases and controls was addressed  *Cross-sectional study*—If applicable, describe analytical methods taking account of sampling strategy | | | | | | | Not applicable | | | | | | | | | | | | | |  | | | | | | | | | | | | |  |  |  |  |  |  |
|  |  |  |  |  | (*e*) Describe any sensitivity analyses | | | | | | | | | | | | | | | | | | | | | | | |  | | |  | | | | | | |  |  |  |  |  |  |
| Results | | | | | | | | | | | | | | | | | | | | | | | | | | | | | | | | | | | | | | |  |  |  |  |  |  |
| Participants | | 13* | | | (a) Report numbers of individuals at each stage of study—eg numbers potentially eligible, examined for eligibility, confirmed eligible, included in the study, completing follow-up, and analysed | | | | | | | | | | 5&8 | | | | | | | | | The study included any acute respiratory illness (runny nose and sore throat) and at least one of the following symptoms: fever, cough, and shortness of breath. All suspected COVID-19 patients who have essential clinical features of acute respiratory illnesses or the most common presenting symptoms of acute respiratory illnesses (cough and dyspnea**),** influenza-like symptoms (fever and myalgia) and known contact history with COVID-19 patients were included in the study, whereas previously known COVID-19 disease patients were excluded from the study. | | | | | | | | | | | | | | |  |  |  |  |  |  |
|  |  |  |  |  | (b) Give reasons for non-participation at each stage | | | | | | | | | | | | | | | | 5 | | | | previously known COVID-19 disease patients were excluded from the study | | | | | | | | |  |  |  |  |  |  |  |  |  |  |  |
|  |  |  |  |  | (c) Consider use of a flow diagram | | | | | | | | | | | | | | | | | | | | | | | |  | | |  | | | | | | |  |  |  |  |  |  |
| Descriptive data | | 14* | | | (a) Give characteristics of study participants (eg demographic, clinical, social) and information on exposures and potential confounders | | | | | | | | | | | | | | | | | 8 | | | From the total of 413 participants, more than half of them were males. The median age was 56 years old, with an interquartile range of 25 years old. The majority of patients were married, 277(67.1%), came from urban areas, 346 (83.8%), and had primary and above-primary educational levels 330 (79.9%). Similarly, the majority of patients reported that they were not smoking cigarettes, 397 (96.1%), regularly using alcohol 338 (81.8%), and chewing Khat in 394 (95.4%) of patients.  Of all participants, 250 (60.5%) were confirmed for SARS-CoV-2 infection. The most commonly reported symptoms among suspected patients were shortness of breath, 396 (95.9%) followed by cough, 385 (93.5%) and loss of appetite, 362(87.7%),  A total of 240 patients (58.1%) were found to have co-morbid chronic health conditions, of whom 138 (57.5%) of them being tested positive for COVID-19 disease. | | | | | | | | | | | | | |  |  |  |  |  |  |
|  |  |  |  |  | (b) Indicate number of participants with missing data for each variable of interest | | | | | | | | | | | | | | | 12-14 & 19-21 | | | | | | | | Indicated in table 2 and table 5 | | | | | | | | | | | | |  |  |  |  |
|  |  |  |  |  | (c) *Cohort study*—Summarise follow-up time (eg, average and total amount) | | | | | | | | | | | | |  | | | | | | | | | | | | | |  | | | | | | |  |  |  |  |  |  |
| Outcome data | | 15* | | | *Cohort study*—Report numbers of outcome events or summary measures over time | | | | | | | | | | | | | |  | | | | | | | | | | | | |  | | | | | | |  |  |  |  |  |  |
|  |  |  |  |  | *Case-control study—*Report numbers in each exposure category, or summary measures of exposure | | | | | | | | | | | |  | | | | | | | | | | | | | | |  | | | | | | |  |  |  |  |  |  |
|  |  |  |  |  | *Cross-sectional study—*Report numbers of outcome events or summary measures | | | | | | | | | | | | | | | | | | | | | | | |  | | |  | | | | | | |  |  |  |  |  |  |
| Main results | | 16 | | | (*a*) Give unadjusted estimates and, if applicable, confounder-adjusted estimates and their precision (eg, 95% confidence interval). Make clear which confounders were adjusted for and why they were included | | | | | | | | | | | | | | | | |  | | | | | | | | | Not applicable | | | | | | | |  |  |  |  |  |  |
|  |  |  |  |  | (*b*) Report category boundaries when continuous variables were categorized | | | | | | | | | | | | | | 11&21 | | | | | | | | | | | ^*^ Normal values; LL, the Lower limit of the normal value; UL, the upper limit of the normal value | | | | | | | | | | | |  |  |  |
|  |  |  |  |  | (*c*) If relevant, consider translating estimates of relative risk into absolute risk for a meaningful time period | | | | | | | | | | | | | | | | | | | | | | | |  | | | It is not relevant. | | | | | | |  |  |  |  |  |  |
| Continued on next page Other analyses | 17 | | Report other analyses done—eg analyses of subgroups and interactions, and sensitivity analyses | | | | | | | | | | | | | | | | | |  | | | | | | Not applicable | | | | | |  |  |  |  |  |  |  |  |  |  |  |  |
| Discussion | | | | | | | | | | | | | | | | | | | | | | | | | | | | | | | | |  |  |  |  |  |  |  |  |  |  |  |  |
| Key results | 18 | | Summarise key results with reference to study objectives | | | | | | | | | | | | | | | 25 | | | | | This study identified that COVID-19 patients had a significantly higher proportion of headache, sore throat, loss of sense of smell, and loss of sense of taste when compared to COVID-19 negative patients. Patients with higher educational status were more likely exposed to COVID-19 because of their mobility. The study also found significant differences in blood analysis, biochemical tests (creatinine and BUN) and enzymatic tests (alkaline phosphatase) at the time of presentation between COVID-19 positive patients and those without COVID-19 disease.  Our findings suggest the need to substantially consider headache, sore throat, loss of sense of smell, and loss of sense of taste as potential clinical diagnostic symptoms for early screening and testing for the country’s context. Similarly, blood, biochemical and enzymatic tests may also be potential diagnostic biomarkers in screening and testing the suspected patients for COVID-19. | | | | | | | | | | | | | | | | | |  |  |  |  |
| Limitations | 19 | | Discuss limitations of the study, taking into account sources of potential bias or imprecision. Discuss both direction and magnitude of any potential bias | | | | | | | | 25 | | | | To improve the testing strategies for COVID-19 that may exclude patients with few symptoms, clinical profiles, laboratory and risk profiles in patients presenting with ARI may also have strong input to design, support, implement and integrate into the health system for better prevention, treatment and management of COVID-19 diseases.  Because of the conflicting results in other studies in terms of clinical profiles, laboratory profiles, and risk factors, we suggest to do further cohort study to address this concern and that will improve for early identification of patients, prioritizing the available resources and optimize the management. | | | | | | | | | | | | | | | | | | | | | | | | | | |  |  |  |
| Interpretation | 20 | | Give a cautious overall interpretation of results considering objectives, limitations, multiplicity of analyses, results from similar studies, and other relevant evidence | | | |  | | | | | | | | | We gave a cautious overall interpretation of results considering objectives | | | | | | | | | | | | | | | | |  |  |  |  |  |  |  |  |  |  |  |  |
| Generalisability | 21 | | Discuss the generalisability (external validity) of the study results | | | | | | 25 | | | | The study also found significant differences in blood analysis, biochemical tests (creatinine and BUN) and enzymatic tests (alkaline phosphatase) at the time of presentation between COVID-19 positive patients and those without COVID-19 disease.  This may represent to improve the testing strategies for COVID-19 that may exclude patients with few symptoms and decrease the utilization of the limited available resource in our setting. | | | | | | | | | | | | | | | | | | | | | | |  |  |  |  |  |  |  |  |  |
| Other information | | |  | | | | | | | | | | | | | | | | | | | | | | | | | | | | | |  |  |  |  |  |  |  |  |  |  |  |  |
| Funding | 22 | | Give the source of funding and the role of the funders for the present  study and, if applicable, for the original study on which the present  article is based | | | | | | | 26 | | | | The research was funded by the St. Paul’s Hospital Millennium Medical College. The funder had no role of the in designing of the study, collection, analysis, and interpretation of data. | | | | | | | | | | | | | | | | | | | | | |  |  |  |  |  |  |  |  |  |

*Give information separately for cases and controls in case-control studies and, if applicable, for exposed and unexposed groups in cohort and cross-sectional studies.

**Note:** An Explanation and Elaboration article discusses each checklist item and gives methodological background and published examples of transparent reporting. The STROBE checklist is best used in conjunction with this article (freely available on the Web sites of PLoS Medicine at http://www.plosmedicine.org/, Annals of Internal Medicine at http://www.annals.org/, and Epidemiology at http://www.epidem.com/). Information on the STROBE Initiative is available at www.strobe-statement.org.
