# Supplementary material for: Epidemiological, clinical and laboratory profile of patients presenting with severe acute respiratory syndrome (SARS-CoV-2) in Ethiopia
Source: PLoS One. 2023 Dec 1;18(12):e0295177. doi: 10.1371/journal.pone.0295177 (PMC10691732; doi:10.1371/journal.pone.0295177)
Supplement: Attachment — Submitted filename: Response Letter.docx [file pone.0295177.s002.docx]

PONE-D-23-08723

Epidemiological, clinical and laboratory profile of patients presenting with severe acute respiratory syndrome (SARS-CoV-2) in Ethiopia: Comparative cross-sectional study

Dear Dr. Gize,

Thank you for submitting your manuscript to PLOS ONE. After careful consideration, we feel that it has merit but does not fully meet PLOS ONE’s publication criteria as it currently stands. Therefore, we invite you to submit a revised version of the manuscript that addresses the points raised during the review process.

Please submit your revised manuscript by Jul 17 2023 11:59PM. If you will need more time than this to complete your revisions, please reply to this message or contact the journal office at [plosone@plos.org](mailto:plosone@plos.org). When you're ready to submit your revision, log on to https://www.editorialmanager.com/pone/ and select the 'Submissions Needing Revision' folder to locate your manuscript file.

Please include the following items when submitting your revised manuscript:

- A rebuttal letter that responds to each point raised by the academic editor and reviewer(s). You should upload this letter as a separate file labeled 'Response to Reviewers'.
- A marked-up copy of your manuscript that highlights changes made to the original version. You should upload this as a separate file labeled 'Revised Manuscript with Track Changes'.
- An unmarked version of your revised paper without tracked changes. You should upload this as a separate file labeled 'Manuscript'.

If you would like to make changes to your financial disclosure, please include your updated statement in your cover letter. Guidelines for resubmitting your figure files are available below the reviewer comments at the end of this letter.

We look forward to receiving your revised manuscript.

Kind regards,

Enoch Aninagyei, PhD
Academic Editor
PLOS ONE

***Response: Dear Enoch Aninagyei, PhD, Academic Editor, PLOS ONE***

***We authors would like to thank for your invitation to submit our revised version of the manuscript, PONE-D-23-08723,***

***Epidemiological, clinical and laboratory profile of patients presenting with severe acute respiratory syndrome (SARS-CoV-2) in Ethiopia: Comparative cross-sectional study, that addresses raised points during the review process.***
 ***We included a point-by-point response within the 'Response to Reviewers' box in the submission system and modifications are highlighted in the track changes of the original manuscript, and we uploaded also the clean or unmarked version of our revised paper without tracked changes.***

***Thank you***

| **S. No** | **Points raised by editors and /or reviewers’** | **Responses to points** |
| --- | --- | --- |
| 1 | Please ensure that your manuscript meets PLOS ONE's style requirements, including those for file naming. | In accordance with the editor’s concern, we have now  Confirmed that our revised manuscript meets PLOS ONE’s style requirements. |
| 2 | Please note that funding information should not appear in any section or other areas of your manuscript | We confirmed that funding information is not appeared in any section of the revised version. |
| 3 | We note that the grant information you provided in the ‘Funding Information’ and ‘Financial Disclosure’ sections do not match.  When you resubmit, please ensure that you provide the correct grant numbers for the awards you received for your study in the ‘Funding Information’ section. | We have now ensured you that the correct grant number which we received in the funding information as “This research was funded by the St. Paul’s Hospital Millennium Medical College. The funder had no role of the in designing of the study, collection, analysis, and interpretation of data. |
| 4 | Your ethics statement should only appear in the Methods section of your manuscript. If your ethics statement is written in any section besides the Methods, please move it to the Methods section and delete it from any other section. Please ensure that your ethics statement is included in your manuscript, as the ethics statement entered into the online submission form will not be published alongside your manuscript. | We have corrected that, our ethics statement of the revised version of the manuscript is written only in the methods section. |
| 5 | Please include captions for your Supporting Information files at the end of your manuscript, and update any in-text citations to match accordingly. Please see our Supporting Information guidelines for more information: http://journals.plos.org/plosone/s/supporting-information. | Thank you for your concern. We have now included the three captions of our supporting Information file at the end of our revised manuscript. |
|  | **Reviewer's Responses to Questions**  **Comments to the Author**  1. Is the manuscript technically sound, and do the data support the conclusions?  The manuscript must describe a technically sound piece of scientific research with data that supports the conclusions. Experiments must have been conducted rigorously, with appropriate controls, replication, and sample sizes. The conclusions must be drawn appropriately based on the data presented.  Reviewer #1: Partly  Reviewer #2: Yes | We would like to thank all reviewers for their critical evaluation and giving such kind of evaluation from our manuscript. |
|  | 2. Has the statistical analysis been performed appropriately and rigorously?  Reviewer #1: Yes  Reviewer #2: No | With all due respect to the reviewer #2, we believe that this point is not correct. The statistical analysis has been performed appropriately and rigorously by our public health professionals (Epidemiologist). |
|  | 3. Have the authors made all data underlying the findings in their manuscript fully available?  Reviewer #1: Yes  Reviewer #2: Yes | Again, authors would like to thank both reviewers for their critical judgment on our manuscript data. |
|  | 4. Is the manuscript presented in an intelligible fashion and written in standard English?  PLOS ONE does not copyedit accepted manuscripts, so the language in submitted articles must be clear, correct, and unambiguous. Any typographical or grammatical errors should be corrected at revision, so please note any specific errors here.  Reviewer #1: No  Reviewer #2: Yes | Reviewer #1 concern is well taken, and any typographical or grammatical errors on our manuscript has corrected in the revised version of manuscript by experts. |
|  | **5. Review Comments to the Author**  Please use the space provided to explain your answers to the questions above. You may also include additional comments for the author, including concerns about dual publication, research ethics, or publication ethics. (Please upload your review as an attachment if it exceeds 20,000 characters) | **Possible responses for reviewers comment and concerns** |
|  | **Reviewer #1:** Good attempt to review and report on COVID-19 cases that were seen in your facility. However, take note of the following observations: 1. The entire manuscript needs grammatical correction. I recommend they consult an editor to review the manuscript and correct the writings before submission. | Thank you very much for your constructive comment. The comment is accepted. As far as possible the revised version of our manuscript grammar is edited by the best English language speaker before submission. |
|  | **Results** 1. The authors indicated a sample of 413 respondents, however, on Table 2, all variables reported had different sample size or denominator. How is this possible for haematological parameters run at the same time? | It is true that the total sample size was 413 as indicated in the socio-demographic table. However, with different reason, all participants might not give their clinical sample to the stated objective of study. Written informed consent was taken, and patients were participated voluntarily. So, we did not urge participants who refuse to give all required clinical samples. |
|  | 2. The authors mentioned co-morbidities that is hypertension and diabetes. These two diseases may not cause the elevation of the haematological parameters like the Neutrophils and enzymematic markers such as ALP, however, other disease conditions like liver disease and other chronic inflammatory conditions may present with similar elevations. Therefore, the authors must indicate which specific chronic conditions or metabolic diseases that were ruled out in the study apart the hypertension and diabetes. | We agree that this is an important area that requires further research, and may not our objective. However, in our study, we have only described participants’ chronic or underlined disease condition, in figure 2. |
|  | **Discussion** 1. The discussion is not cogent; there are contradictory statements and unfounded assumptions. 2. On paragraph 2, the assumption made on the presence of comorbidity and ARI increases the positivity of COVID-19 should be further explain since the assumption does not clearly indicate specifically true positivity reaction or false positivity reaction. | We acknowledge that our manuscript might not have been indicated specifically true positivity reaction or false positivity reaction. But we have described about our participants disease condition saying that “total of 240 patients (58.1%) were found to have co-morbid chronic health conditions, of whom 138 (57.5%) of them being tested positive for COVID-19 disease”, stated in **COVID-19 status: Signs and symptoms** section of our manuscript. |
|  | **Conclusion** The conclusion is not precise and concise, rather a repetition of the results and aspects of the discussion. Consider rewording the paragraph 2 as the conclusion of the study. However, the clinical diagnostics symptoms reported is a general knowledge that has been reported by several studies. Given your findings, are you recommending that will diagnosis and treatment of COVID-19 patients could be done based on symptoms rather than PCR test? | The comment is accepted. We have modified the conclusion section on paragraph 2 and the entire section of conclusion based on your constructive comment. |
|  | **Reviewer #2:** This article aimed to compare epidemiological, clinical, and laboratory profiles of patients presenting with acute respiratory syndrome illness in Addis Ababa Ethiopia. The specific research question is whether the epidemiological, clinical, and laboratory profile data of COVID-19 patients managed in low-resource settings might be quite different from findings from developed countries. | The comment/concern is accepted. There is the scarcity of literature in resource limited country, and as you highlighted the specific research question is whether the epidemiological, clinical, and laboratory profile data of COVID-19 patients managed in low-resource settings might be quite different from findings from developed countries. |
|  | The study reports the results of N=413 patients who had acute respiratory illnesses and were suspected of having COVID-19 disease. A total of 250 (60.5%) patients tested positive for COVID-19 disease. COVID-19 patients were less likely to use an alcohol-based method of hand washing, and a higher proportion of headaches, sore throat, and loss of sense of taste. The study further found that Neutrophil, creatinine, and alkaline phosphatase were significantly higher in COVID-19 patients.  The strength of the study was that a lot of variables were collected, and a well-written manuscript. In addition, the study aims at comparing epidemiological, clinical, and laboratory profiles of patients presenting with acute respiratory syndrome illnesses. This research team is in a unique position to address this question. The results are thus of potentially great interest, but there are some issues with the manuscript that need to be addressed. | It is true that our finding stated that COVID-19 patients were less likely to use an alcohol-based method of hand washing, and a higher proportion of headaches, sore throat, and loss of sense of taste, and the study further found that Neutrophil, creatinine, and alkaline phosphatase were significantly higher in COVID-19 patients. We authors appreciated the reviewer feedback given our manuscript. |
|  | **Major Comments** Line 282-283: Authors should run a logistic regression to test for association. | Our study was titled with “Epidemiological, clinical and laboratory profile of patients presenting with severe acute respiratory syndrome (SARS-CoV-2) in Ethiopia: Comparative cross-sectional study”. It was not a study identifying the associated factors. And regarding the statistical analysis we have stated that, a descriptive analysis was done to see the characteristics of the study subjects. Continuous variables were dichotomized when clinically relevant. We estimated the mean and standard deviation for normally distributed continuous variables and used t-tests to see if there were any differences between the COVID-19 positive and COVID-19 negative patients. We used the chi-square test or Fisher's exact test to assess differences between groups for categorical and dichotomous data, in the data entry and analysis section of the manuscript. So, With all due respect to the reviewer, we believe that logistic regression to test for association was not important for the stated objective of the study. |
|  | **Minor Comments** **Abstract** Line 33: delete ‘was’ (the study aimed to compare). | The comment is accepted. Based on the comment our revised manuscript is corrected. |
|  | Line 34: Add ‘of’ (clinical and laboratory profiles of patients). | The comment is accepted. Based on the comment our revised manuscript is corrected. |
|  | Line 38: Add ‘a’ (Using a structured questionnaire) | The comment is accepted. Based on the comment our revised manuscript is corrected. |
|  | Line 41: Replace (to see their laboratory profiles) with ‘for laboratory profile analysis’. | The comment is accepted. Based on the comment our revised manuscript is corrected. |
|  | Line 45: delete ‘were’ (participants tested positive) | The comment is accepted. Based on the comment our revised manuscript is corrected. |
|  | **Methods** Line 111: what about those who are 18 years and below? | Participants who were under 18 years were not our study subject. They were not included in our inclusion criteria. |
|  | Line 113: A suspected case is based on meeting one or more of either clinical or epidemiological link criteria. It is not clear why, according to the authors, patients were tested irrespective of whether they met the criteria or not. | Sorry for the mistake, by now it is corrected as “Suspected patients were tested for SARS-CoV-2 if they met COVID-19 symptoms clinically or laboratory testing criteria of the National Ethiopian Public Health Guideline”, in the revised manuscript. |
|  | Line 117: The sentence should be paraphrased. | The comment is accepted. Based on the comment our revised manuscript is paraphrased. |
|  | Line 134: Per the calculation, the minimum sample size must be 423. | Thank you. Sorry for the mistake, by now it is corrected as a minimum sample size to be 423 and not 422 in the revised manuscript. |
|  | Line 142: delete ‘an.’ | The comment is accepted. Based on the comment our revised manuscript is corrected. |
|  | Line 149: This statement contradicts that of line 113. The authors must clarify this. | The comment is accepted. Based on the comment we clarified by no in our revised manuscript in the line 113. |
|  | Line 156: Provide a reference for the protocol or the standard operating procedure. | Thank you for your constructive comment. However, we have already cited our study protocol as a reference “24” in the main document and listed as “FMOH. National Comprehensive COVID-19 Management Handbook. Addis Ababa; 2020”, in the reference list. |
|  | Line 182: With the biochemical and enzymatic tests, the blood was collected into which tube since an inappropriate tube can lead to wrong results. E.g., sodium fluoride (NaF) tubes and serum separation tubes (SST) | Thank you again. The comment is taken and modification is done on the revised manuscript. For your information we have used serum separation tubes (SST). |
|  | Line 192-193:= 215-216 Authors should state exactly what was done to prevent or minimize pre-analytical, analytical, and post-analytical errors. Specimen collection, handling, storage including temp, Assays used, quality control testing, and reliability of assays. | The comment is accepted.  For laboratory analysis pre-analytical such as identifying the right patient and laboratory sample type and time, sample handling, patient and, material preparations, checking of quality control; in the analytical phase we follow starting samples have been logged into the lab. This phase is comprised of the lab diagnostic and testing procedures and instrument analysis. In the post-analytical stages, we monitored correctly the result dispatch, and verification of the results of the laboratory work was done to maximize the quality data that were proven in the Standard Operating Procedures (SOPs) of the laboratory. |
|  | **Results**  Line 211: Indicate the figure. | The comment is accepted. Based on the comment, we have corrected on the revised manuscript. |
|  | Line 216: Authors should re-categorize the groups i.e., Occupation. I think some health professionals, as well as drivers, are Government employees. -Education: The word illiterate is too offensive. Replace with no formal education. College and above: replace with tertiary. | The comment is accepted. Based on the comment, we have modified on the revised manuscript. |
|  | Line 231: Authors must be consistent in reporting. Just use the percentages | The comment is accepted. By now we have used percentages consistently in the revised manuscript. |
|  | Line 260: Use a ‘differential blood count.’ | The comment is accepted. We have modified the revised manuscript based on the comment as “Differential blood count” |
|  | **Discussion**  Line 294-295: It is not clear which respiratory tract infections apart from Covid-19, that, the authors compare the clinical, laboratory, and other variables with. | The comment is well taken. By now we have removed the phrase “Without a control group and limited COVID-19 test availability, we cannot ensure whether COVID-19 presents differently from other types of respiratory diseases” form the revised version of the manuscript. |
|  | Line 296-297: the study site is a referral and treatment center. Most patients are likely to test positive for Covid-19. Moreover, some might be severely ill and have received some form of treatment before being referred to the facility. This can lead to bias in the study | The comment is well taken. It is true that the hospital is a referral specialized hospital in Addis Ababa. Patients presenting with SARS-CoV-2 came to the center from various corners of the country outside of the capital city of Ethiopia. Additionally, the study included any acute respiratory illness (runny nose and sore throat) and at least one of the following symptoms: fever, cough, and shortness of breath, whereas previously known COVID-19 disease patients were excluded from the study, this concern incorporated as a limitation of the study. |
|  | 6. PLOS authors have the option to publish the peer review history of their article ([what does this mean?](https://journals.plos.org/plosone/s/editorial-and-peer-review-process#loc-peer-review-history)). If published, this will include your full peer review and any attached files.   If you choose “no”, your identity will remain anonymous but your review may still be made public.  **Do you want your identity to be public for this peer review?** For information about this choice, including consent withdrawal, please see our [Privacy Policy](https://www.plos.org/privacy-policy).  Reviewer #1: **Yes:** Comfort Dede Tetteh  Reviewer #2: No | We thank all editors and reviewers participated to review this manuscript. We authors have no any problem weather it was anonymously reviewed or not. |
