# Supplementary material for: Epidemiological, clinical and laboratory profile of patients presenting with severe acute respiratory syndrome (SARS-CoV-2) in Ethiopia
Source: PLoS One. 2023 Dec 1;18(12):e0295177. doi: 10.1371/journal.pone.0295177 (PMC10691732; doi:10.1371/journal.pone.0295177)
Supplement: Attachment — Submitted filename: Response to reviwers_Aman_R2.docx [file pone.0295177.s003.docx]

Top of Form

**View Letter**

Bottom of Form

| Date: | Aug 02 2023 12:35PM |
| --- | --- |
| To: | "Addisu Gize" konjoaddisu@gmail.com |
| From: | "PLOS ONE" plosone@plos.org |
| Subject: | PLOS ONE Decision: Revision required [PONE-D-23-08723R1] |

PONE-D-23-08723R1

Epidemiological, clinical and laboratory profile of patients presenting with severe acute respiratory syndrome (SARS-CoV-2) in Ethiopia: Comparative cross-sectional study

PLOS ONE

Dear Dr. Gize,

Thank you for submitting your manuscript to PLOS ONE. After careful consideration, we feel that it has merit but does not fully meet PLOS ONE’s publication criteria as it currently stands. Therefore, we invite you to submit a revised version of the manuscript that addresses the points raised during the review process.

***Response: We authors would like to thank for your invitation to submit our revised version of the manuscript, PONE-D-23-08723R1***

***Epidemiological, clinical and laboratory profile of patients presenting with severe acute respiratory syndrome (SARS-CoV-2) in Ethiopia: Comparative cross-sectional study. In the revised version of the manuscript, we addressed points raised during the review process.***

**ACADEMIC EDITOR:**

Title: Since you did not recruit participants without SARS-CoV-2, delete Comparative cross sectional study from the title

***Response: The comment is accepted. By now we have removed “comparative cross sectional study from our revised manuscript title.***

Line 41: specify the laboratory profiles you analyzed

***Response:* *The comment is accepted. In the revised manuscript we have specified as “hematological tests like; white blood cell count, hematocrit, and platelet count; and biochemical and enzymatic tests like; aspartate transaminase (AST), creatinine, etc are mentioned/specified (line 42-44, within track change file of the abstract section revised manuscript).***

Line 42: Revise …p. value less than 0.05 were to p-values less than 0.05… or p-values < 0.05

***Response: Thank you! The comment is accepted. Based on the comment, we have corrected it (line 45, within track change file of the abstract section revised manuscript).***

Line 44: SARS-CoV-2 and not SARS- CoV-2

***Response: The comment is accepted. Based on the comment, we have corrected it (line 47, within track change file of the abstract section revised manuscript).***

Line 49: neutrophil and not Neutrophil

***Response: The comment is accepted. Based on the comment, we have corrected it (line 52, within track change file of the abstract section revised manuscript).***

Line 50: Show mean and SD values instead of %. Also indicate the p-values so confirm significance

***Response: Thank you for the comment. Since each laboratory-related factor has a cut point where it may be classified as normal range, if it is in the normal, and if it is abnormal we categorized as abnormally high values (above the normal range), or low values (below the normal range), we analyzed the participant laboratory values as categorical variables(low, normal and high values). Examples include creatinine, alkaline phosphatase, and others. This categorical classification is clinically significant and applicable to treat the participants rather than comparing mean and SD laboratory values.***

line 54: do not capitalize Creatinine, Alkaline. Are trying to conclude that elevation of these markers confirms COVID-19? Please revise this statement

***Response: Based on the comment we have revised it (line 58 within track change file of the abstract section revised manuscript).***

Lines 60/70: Define all abbreviations (COVID-19, CDC) on first mention

***Response: Thank you for your constructive comments. Based on the comment we have revised it (line 72 and 83 within track change file of the introduction section revised manuscript).***

Lines 68/69: Try expressing these huge number in millions and billions for ease of appreciation

***Response: Thank you for your constructive comments. Based on the comment we have revised it. (Lines 81 and 82 within track change file of the introduction section revised manuscript)***

Lines 82-84: Were these parameters elevated or reduced?

***Response: Thank you for your constructive comments. They are elevated during infection (Line 96 within track change file of the introduction section revised manuscript)***.

Lines 131/132: Because we had no previous proportion of positivity during the study period, we took the positivity rate of RT-PCR COVID-19 to be 50%...refer readers to previous publication where this assumption was used

***Response: With all due respect to the editors’, we believe that this point is not correct***; we had no previous study means, it is recommended by the statistics science to use the formula (50% proportion) to calculate the sample size determination for the current study.

Lines 129-36: indicate the formula used to determine the sample size

***Response: The formula is indicated below (written on lines 152-158 within track change file of the method section revised manuscript).***

n = (z^α^/_2_)^2^ p (1-p)	(1.96)^2^ 0.5(1-0.5) = 384 study subjects.

d^2^	(0.05)^2^

Where: n = minimum sample size required,

P = estimated proportion of patients with *SARS-CoV-2* in the study population, and taking 10% non-response rate, the final sample size become 423 participants.

d= the margin of sample error, z^α^/_2=_ the standard normal variable at 1-^α^_/2_ confidence level and we used consecutive sampling technique was used to select the study population.

Line 159: indicate the source of the Da’an Gene Corporation. Does the kit isolate NA or RNA? Specify

***Response: It is sourced from Guangdong, China, and can isolate RNA (Line 185 within track change file of the method section revised manuscript).***

Lines 169/170: indicate the rtqPCR equipment used

***Response: A quantitative PCR instrument is a machine that amplifies and detects DNA or RNA. In the case of SARS-CoV-2, the rtqPCR combines the functions of a thermal cycler and a fluorimeter, enabling the process of quantitative PCR (Lines 198-200 of within track change file of the method section revised manuscript).***

Line 176: vacutainer

***Response: The comment is accepted. Based on the comment we have corrected (written on line 206 within track change file of the method section revised manuscript).***

Lines 175/182: Indicate the volume of blood samples collected into each tube

***Response: The comment is accepted. Based on the comment we have corrected the volume of blood taken from each study subject as 5ml (written on line 205 within track change file of the method section revised manuscript).***

Line 185: The instrument was fully automated and can do up to 60 assays like hemoglobin A1C (HBA1C), Alanine Aminotransferase (GPT), etc…statement not necessary

***Response: Thank you for your constructive comment. Now we have removed the unnecessary statement from the revised manuscript.***

Line 191: indicate the Westgard rule used to assess the validity of the biochemistry values

***Response:*** ***We used Westgard rules to assess the validity of the biochemistry values and the rule recommends tolerating only 12s i.e., one measurement exceeds 2 standard deviations either above or below the mean of the reference range (written on line 230-232 within track change file of the method section revised manuscript).***

Line 193/99: sentence too long. Revise

***Response: Thank you for your constructive comment. Now we have revised it.***

Line 211: include ethics approval reference number

***Response:*** ***Thank you. The comment is taken and by now we have added ethics approval reference number to the revised manuscript (written on line 245 within track change file of the method section revised manuscript).***

Line 226: More than half of them were males.. add number (%)

***Response: The comment is taken and edited as; n=244(59.1%) (Written on line 279 within track change file of the result section revised manuscript)***

Line 237: change figure 1 to (figure 1) same as line 241

***Response: The comment is accepted and edited as (figure 1) and (figure 2) in lines 289 and 293*** ***within track change file of the method section revised manuscript***

Lines 243/44: what do you mean by higher, lower and normal values? Indicate actual mean values and p-values to show sig differences

***Response: With all due respect to the editor, this point is not correct.***

***Every laboratory test has its own reference range. For example the normal reference ranges for hemoglobin count for adult population described in different book as “13.6-17.5 g/dL” of blood. This means, below the normal limit (˂13.6g/dL) i.e., we will call it lower or abnormally low result, clinically leads to anemia, and also if it is above the normal limit .i.e., greater than 17.5g/dL, also indicate abnormally high hemoglobin level of the patient. So, we have to classify our study participants based on these reference ranges as how many of the participants are in the normal reference ranges. i.e., 13.6-17.5g/dL for hemoglobin laboratory results as example given above, and other laboratory profiles too based on their normal reference ranges at the time of their presentation for diagnosis. Regarding to the p-value and significance, it is listed in the table 5 for each laboratory parameter.***

Table 2: I am not sure the table is well presented. Which population does the normal values you indicated refer to? Of the 413 cases, 250 and 163 were positive and negative respectively. Compare the mean blood parameters between the positive and negative cases. In its current form, it doesn’t bring out a good outcome of the intended analysis.

***Response: This table 2 is the laboratory test results of all patients at the time of presentation. And categorized as how many of the participants were in the normal reference range on each laboratory test results, how many them were below the reference range (low) and how many of them were elevated (high) their result from each reference range. For example from this table in the case of white blood cell count (n=319 participants) at the time of presentation, of which 14(4.4%) of them were below the normal reference range level, lower than lower limit (lower than the LL i.e., less than 3.4-10 x109/L, we will call it leukopenia, and 119(37.3%) had greater than 10 x109/L white blood cell count, we will call it leukocytosis or abnormally elevated number of white blood cells whereas the majority category of the participants, 186(58.3%) had normal values (fall in the normal reference interval), ( table 2).***

Line 258: You use college in the test and tertiary school in table 3, please be consistent

***Response: The comment is accepted. By now we have consistent having tertiary school (in the line 317 within track change file of the result section revised manuscript***

Lines 258-60: patients with a positive COVID-19 test 259 were less likely to use the only alcohol-based method of hand washing (12.5% vs 87.5%, P<0.05),… how did you determine the less likelihood?

***Response: Thank you for the valuable comment. Since we conducted the analysis using chi-square test, we can’t use the word less likely and we have corrected this in the manuscript using the chi squire test interpretation (line 317-321).***

Table 3, under occupation, why is the frequency for government in parenthesis?

***Response: Sorry for the mistake. By now we have removed it.***

Table 3, why did you use fisher test for ‘Daily rubbing hands’ when all the frequencies were more than 5

***Response: Thank you so much for your valuable comment. We have re-run the analysis again and the test fits for chi-square and not for fisher exact test. It is corrected accordingly.***

Table 3: was Method of hand washing analyzed with chi or fisher exact?

***Response: Thank you for your valuable comment. We took the chi squire test p-value; however, it must be fisher exact test. We have corrected accordingly.***

Lines 265-68: better to write p=0.001 instead of P=0.001. Revise here and elsewhere

***Response: Thank you for your constructive comment. Now we have corrected.***

Table 5: T-test is more appropriate to analyze Table 5

***Response: Thank you for your comment. Clinically it is very important to classify laboratory results or values as low, normal and high. Hence, we have considered the laboratory profile as a categorical covariate variable (categorized as below the normal reference interval, normal reference interval and above reference interval). Also the data was analyzed using the chi squire and fisher exact test statistics.***

Line 301: Obviously these changes will affect the discussion. Please revise the discussion after effecting these changes.

***Response: Yes, thank for your concern and constructive comments. By now we have gone through in all section of the manuscript (discussion, conclusion and recommendation).***

Please submit your revised manuscript by August 16 2023 11:59PM. If you will need more time than this to complete your revisions, please reply to this message or contact the journal office at [plosone@plos.org](mailto:plosone@plos.org). When you're ready to submit your revision, log on to https://www.editorialmanager.com/pone/ and select the 'Submissions Needing Revision' folder to locate your manuscript file.

Please include the following items when submitting your revised manuscript:

- A rebuttal letter that responds to each point raised by the academic editor and reviewer(s). You should upload this letter as a separate file labeled 'Response to Reviewers'.
- A marked-up copy of your manuscript that highlights changes made to the original version. You should upload this as a separate file labeled 'Revised Manuscript with Track Changes'.
- An unmarked version of your revised paper without tracked changes. You should upload this as a separate file labeled 'Manuscript'.

***Response: We included a point-by-point response within the 'Response to Reviewers' box in the submission system and modifications are highlighted in the track changes of the original manuscript, and we uploaded also the clean or unmarked version of our revised paper without tracked changes, file labeled as ‘Manuscript’.***

If you would like to make changes to your financial disclosure, please include your updated statement in your cover letter. Guidelines for resubmitting your figure files are available below the reviewer comments at the end of this letter.

***Response: No need of change about our manuscript regarding to financial disclosure***

We look forward to receiving your revised manuscript.

Kind regards,

Enoch Aninagyei, PhD
Academic Editor
PLOS ONE

***Response: Thank you Enoch Aninagyei, PhD, Academic Editor. We have revised based on the raised points.***

Reviewers' comments:

Reviewer's Responses to Questions

**Comments to the Author**
1. If the authors have adequately addressed your comments raised in a previous round of review and you feel that this manuscript is now acceptable for publication, you may indicate that here to bypass the “Comments to the Author” section, enter your conflict of interest statement in the “Confidential to Editor” section, and submit your "Accept" recommendation.

Reviewer #1: All comments have been addressed

***Response: Thank you for your scientific contribution to improve our manuscript.***

2. Is the manuscript technically sound, and do the data support the conclusions?

The manuscript must describe a technically sound piece of scientific research with data that supports the conclusions. Experiments must have been conducted rigorously, with appropriate controls, replication, and sample sizes. The conclusions must be drawn appropriately based on the data presented.

Reviewer #1: Yes

***Response: Thank you.***

3. Has the statistical analysis been performed appropriately and rigorously?

Reviewer #1: Yes

***Response: Thank you.***

4. Have the authors made all data underlying the findings in their manuscript fully available?

The [PLOS Data policy](http://www.plosone.org/static/policies.action#sharing) requires authors to make all data underlying the findings described in their manuscript fully available without restriction, with rare exception (please refer to the Data Availability Statement in the manuscript PDF file). The data should be provided as part of the manuscript or its supporting information, or deposited to a public repository. For example, in addition to summary statistics, the data points behind means, medians and variance measures should be available. If there are restrictions on publicly sharing data—e.g. participant privacy or use of data from a third party—those must be specified.

Reviewer #1: Yes

***Response: Thank you.***

5. Is the manuscript presented in an intelligible fashion and written in standard English?

PLOS ONE does not copyedit accepted manuscripts, so the language in submitted articles must be clear, correct, and unambiguous. Any typographical or grammatical errors should be corrected at revision, so please note any specific errors here.

Reviewer #1: Yes

***Response: Thank you.***

6. Review Comments to the Author

Please use the space provided to explain your answers to the questions above. You may also include additional comments for the author, including concerns about dual publication, research ethics, or publication ethics. (Please upload your review as an attachment if it exceeds 20,000 characters)

Reviewer #1: Thank you for considering the comments suggested and addressing them accordingly to improve on the manuscript.

***Response: Thank you.***

7. PLOS authors have the option to publish the peer review history of their article ([what does this mean?](https://journals.plos.org/plosone/s/editorial-and-peer-review-process#loc-peer-review-history)). If published, this will include your full peer review and any attached files.


If you choose “no”, your identity will remain anonymous but your review may still be made public.


**Do you want your identity to be public for this peer review?** For information about this choice, including consent withdrawal, please see our [Privacy Policy](https://www.plos.org/privacy-policy).

Reviewer #1: **Yes:** Comfort Dede Tetteh

***Response: Dear Comfort Dede Tetteh, Thank you so much.***

*In compliance with data protection regulations, you may request that we remove your personal registration details at any time.* [*(Remove my information/details)*](https://www.editorialmanager.com/pone/login.asp?a=r)*. Please contact the publication office if you have any questions.*

Top of Form

Bottom of Form
